# A QUALITATIVE STUDY OF THE WRITINGS OF LONELY OLDER CHRONICALLY ILL ADULTS LIVING IN APPALACHIA

**DOI:** 10.1093/geroni/igad104.0073

**Published:** 2023-12-21

**Authors:** Laurie Theeke, Maritza Dowling, Lashawn Hutto, Michelle Odlum

**Affiliations:** The George Washington University, Washington, District of Columbia, United States; The George Washington University, Washington, District of Columbia, United States; The George Washington University, Washington, District of Columbia, United States; The George Washington University, Washington, District of Columbia, United States

## Abstract

Loneliness is a well-known growing public health risk that affects a large proportion of our aging population. The perspectives of older adults experiencing loneliness are essential to identify areas of potential support and intervention but have been understudied. We collected writings from older adults who participated in LISTEN intervention studies focusing on belonging, relationships, getting out in the community, challenges of loneliness, and finding meaning. Thirty-three community dwelling elders (81% female; mean age=71.77, SD=10.09 years) with multiple chronic conditions (mean number=2.63, SD=1.55) who reported high loneliness scores in the UCLA Loneliness Scale (mean=52.27; SD=8.302) contributed 50 minutes of writings or recordings (for those who were unable to write). These were accomplished in 10-minute increments during five weekly sessions of LISTEN. Thematic content analysis was conducted on the writings and the transcribed recordings. Five themes emerged: Lost Connections and Self-isolation, Empty Relationships, Life is Upside Down and Can’t Be Controlled, Doing Something is Better than Doing Nothing, and Loneliness is Haunting and Relentless. Participants admitted not reaching out to others, worried about being a burden, feeling lonely while with family, and ‘not being in the loop’. The downside of life was described as an uncertainty for the future and imminent functional decline. They described how loneliness ‘screams’ at you and is ‘always lurking’. Activities that ameliorated loneliness included painting, reading, crafting, puzzles, television, and faith activities. These findings will inform the refinement of the LISTEN intervention and the design of new interventions for loneliness.

